# Case of an unreported genetic variant of salt losing 3-β-hydroxysteroid dehydrogenase deficiency

**DOI:** 10.1093/omcr/omab021

**Published:** 2021-05-24

**Authors:** Einas H Alkhatib, Stacie D Adams, Emily R Miller

**Affiliations:** 1 Department of Pediatrics, Michigan State University/Helen DeVos Children’s Hospital, Grand Rapids, Michigan, Grand Rapids, MI, USA; 2 Division of Pediatric Neurosciences, Section of Pediatric Biochemical Genetics, Michigan State University/Helen DeVos Children’s Hospital, Grand Rapids, Michigan, Grand Rapids, MI, USA; 3 Division of Pediatric Endocrinology, Michigan State University/Helen DeVos Children’s Hospital, Grand Rapids, Michigan, 100 MI St NE, Grand Rapids, MI, USA

**Keywords:** pediatrics, endocrinology, congenital adrenal hyperplasia, adrenal, genetics

## Abstract

Salt losing 3-β-hydroxysteroid dehydrogenase deficiency (HSD3B2) is a rare form of congenital adrenal hyperplasia, seen in <0.5% of cases. We present a 7-year-old male diagnosed with HSD3B2 deficiency, not identified by state newborn screen, due to a novel variant identified in the HSD3B2 gene (c.694C > G; p.His232Asp). This patient was referred to pediatric endocrinology and pediatric biochemical genetics following a fourth hospitalization for emesis and electrolyte derangements including hyponatremia, hyperkalemia, ketoacidosis and hypoglycemia. Endocrinology evaluation yielded elevated 17-hydroxyprogesterone (17-OHP), 17-hydroxypregnenolone (17-OHPreg), dehydroepiandrosterone and adrenocorticotropic hormone (ACTH). ACTH stimulation test indicated flat response. Sequencing of the HSD3B2 revealed a pathogenic variant inherited in trans with the novel c.694C > G (p.His232Asp) variant. The patient was started on daily glucocorticoid and mineralocorticoid replacement and has since had no further adrenal crises.

## INTRODUCTION

Congenital adrenal hyperplasia (CAH) results from deficiency of an enzyme that forms [1] cortisol from cholesterol [2]. Left untreated, CAH may lead to life-threatening adrenal crises including hypoglycemia, electrolyte abnormalities and hemodynamic instability [1].

**Figure 1:**
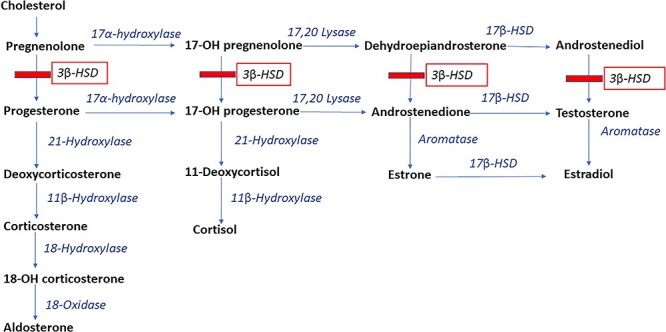
Adrenal steroidogenesis pathway showing effects of 3β-HSD deficiency.

3-β-hydroxysteroid dehydrogenase (3βHSD) deficiency is a rare form of CAH caused from defects in HSD3B2 gene located at chromosome 1p12 expressed primarily in the adrenal glands and gonads [1, 3]. Normally, 3βHSD catalyzes the conversion of pregnenolone to aldosterone, 17-hydroxypregnenolone (17OHPreg) to cortisol and dehydroepiandrosterone (DHEA) to androstenedione. Defects in HSD3B2 block conversion of precursor hormones leading to a build-up of metabolites [2] (Fig. 1).

Diagnosis is made by a ∆5-17OHPreg level above 3300 ng/dL [2]; however, it can also result in elevated renin, 17-hydroxyprogesterone (17-OHP), DHEA and/or urinary ∆5-OHP, as well as high testosterone in females. Newborn screening in the USA assesses for elevated 17-OHP levels above referral cut-off (<19 mg/mL) [1]. Those with the classic form of 3βHSD deficiency develop salt wasting, hyperkalemic acidosis and hypoglycemia. Males may present with signs of under-virilization, including hypospadias, while females often have phenotypic genitalia [1]. Patients can also present with ambiguous genitalia without salt wasting, or even non-classically with premature pubarche, hirsutism and irregular menses in females. CAH-related illnesses may lead to adrenal crises and urgent hospitalization [2].

A c.35G > A founder mutation of the HSD3B2 gene is present in the Old Order Amish; however, there are more than 40 pan-ethnic pathogenic variants identified. Here, we present a 7-year-old male diagnosed with 3βHSD deficiency with two distinct genetic mutations, one of them previously unreported.

## CASE REPORT

A 7-year-old male presented with a fourth episode of hyponatremia, hyperkalemia, metabolic acidosis and hypoglycemia.

Birth history was significant for penoscrotal hypospadias and chordee. State newborn screen was normal, and karyotype was 46 XY. The ultrasound revealed absent Mullerian structures. He was treated with hypospadias repair; however, cause of ambiguous genitalia was not evaluated further. Past medical, surgical and family histories were otherwise negative.

His first episode concerning for adrenal crisis was at 3 years of age when he presented with fever, prolonged emesis, hyponatremia, hyperkalemia, metabolic acidosis and hypoglycemia. He required brief hospitalization for correction of electrolytes and dehydration. At 5 years of age, he had two similar episodes with electrolyte derangements. After the third episode, evaluation included normal upper gastrointestinal tract radiography, lactic acid, acylcarnitine profile and ammonia. Random cortisol level was 20 mcg/dL during illness, however, was not repeated when well.

At 7 years of age, he presented with persistent non-bloody, non-bilious emesis. Labs were notable again for hyponatremia, hyperkalemia, hypoglycemia, ketonuria and metabolic acidosis (Table 1). Vitals showed heart rate 152 beats per minute, temperature 38.2°C, and blood pressure 121/70 mmHg. He appeared mildly dehydrated but otherwise well perfused. He received dextrose 25% (2 mL/kg) bolus for correction of hypoglycemia and three normal saline boluses for correction of his dehydration. He was admitted for correction of electrolytes and dehydration with 0.9% normal saline infusion with 5% dextrose. He was discharged home with outpatient referral to pediatric endocrinology.

**Table 1:** Labs at presentation during fourth episode of vomiting

	Lab value	Reference range
Sodium	126 mmol/L	134–146 nmol/L
Potassium	5.6 mmol/L	3.4–5.0 nmol/L
Chloride	95 mmol/L	98–112 mmol/L
Blood glucose	46 mg/dL	60–99 mg/dL
Urine ketones	80 mg/dL	Negative
Venous pH	7.25	7.32–7.42
Venous pCO2	26.1 mm Hg	40.0–40.0 mm Hg
Serum HCO3	7 mmol/L	21–29 mmol/L

Outpatient endocrinology evaluation included ACTH stimulation testing. Cosyntropin 250 micrograms was administered IV, and adrenal steroid hormones were evaluated at baseline and 60 minutes following injection. Stimulated profile was consistent with 3βHSD deficiency (Table 2). Cortisol level demonstrated a flat response (Table 3).

**Table 2:** Endocrinology lab evaluation 60 minutes following Cosyntropin 250 mcg IV

	Lab value	Reference range
17 OH progesterone	308 ng/dL	<91 ng/dL
17OH pregnenolone	5241 ng/dL	10–186 ng/dL
DHEA	970 ng/dL	<111 ng/dL
ACTH	76 pg/mL	10–60 pg/mL

**Table 3:** Serum cortisol level at baseline and 60 minutes following ACTH administration

	Lab value	Reference range
Prestimulation cortisol	13 μg/dL	3–21 μg/dL
Poststimulation cortisol	14 μg/dL	20–31 μg/dL

Genetic testing was performed at Invitae Labs. Reproductive carrier sequencing and deletion/duplication analysis of the HSD3B2 gene initially revealed two heterozygous variants of uncertain significance (VUS). Subsequent parental testing confirmed that the variants were inherited in trans, with mutations on the same gene, but the opposite allele. The maternally inherited c.518 T > G (p.Leu173Arg) variant has a population significance of 0.003%. It was reported previously in one patient as VUS and was later re-classified as pathogenic [4]. The paternally inherited c.694C > G (p.His232Asp) has not been previously reported, but it is a good candidate for pathogenicity. Thus, the presence of these two variants identified in trans in a patient clinically diagnosed with 3βHSD is considered diagnostic of 3βHSD deficiency.

The patient was started on hydrocortisone 11 mg/m^2^ daily and fludrocortisone 0.05 mg twice daily. In case of future illness, the family was taught oral and injectable stress-dose administration. On follow-up, he is doing well, with no additional adrenal crises.

## DISCUSSION

We report a case of salt-wasting CAH secondary to 3βHSD deficiency in a male patient who did not present until childhood. We suggest pathogenicity of a novel, paternally inherited c.694C > G (p.His232Asp) variant identified in the HSD3B2 gene.

3βHSD deficiency has a variable phenotypic presentation, classified as salt wasting, non-salt wasting or non-classic. The severe, salt-wasting phenotype is associated with frameshift mutations, in-frame deletions and nonsense mutations of the HSD3B2 gene, which causes a lack of 3βHSD enzymatic activity [1, 5]. These infants typically present in the first months of life with adrenal crisis, which can be fatal if untreated. The non-salt-wasting and non-classical phenotypes are attributed to missense mutations of the HSD3B2 gene associated with residual but diminished enzymatic activity. In contrast, patients with these forms may have a delay in presentation and diagnosis.

This patient demonstrates that variable genotypes of 3βHSD are associated with an array of clinical presentations. Although he presents with classical CAH, it is of particular interest that his first episode of salt-wasting crisis occurred after 3 years of age, including no crisis during his hypospadias surgical repair. Given the relation between genotype and degree of enzymatic activity, this suggests he has a small but inadequate degree of residual activity that afforded some protection in early life. However, future functional studies or identification of this variant in others would help elucidate this point. Therefore, it is beneficial to continue genotypic mapping of patients who present clinically with 3βHSD deficiency to identify novel variants in the HSD3B2 gene and further understand the correlation between genotype and phenotypic presentation.

To conclude, early diagnosis and of CAH is crucial. As newborn screening is unlikely to detect 3βHSD deficiency, the identification of novel pathogenic variants is important to aid the diagnosis of affected individuals through molecular testing.
